# Pain as the fifth vital sign—A comparison between public and private healthcare systems

**DOI:** 10.1371/journal.pone.0259535

**Published:** 2021-11-03

**Authors:** Daniel Humberto Pozza, Luís Filipe Azevedo, José Manuel Castro Lopes

**Affiliations:** 1 Department of Biomedicine, Faculty of Medicine, University of Porto, Porto, Portugal; 2 Department of Histology, Faculty of Nutrition and Food Sciences, University of Porto, Porto, Portugal; 3 Institute for Research and Innovation in Health-I3s, University of Porto, Porto, Portugal; 4 Department of Community Medicine, Information and Health Decision Sciences (MEDCIDS), Faculty of Medicine, University of Porto, Porto, Portugal; 5 Centre for Health Technology and Services Research (CINTESIS), University of Porto, Porto, Portugal; University of Copenhagen: Kobenhavns Universitet, DENMARK

## Abstract

**Background:**

The assessment of pain as the fifth vital sign (P5VS) is of paramount importance since it leads to the management of undertreated pain, consequently reducing suffering, readmissions and emergency department visits after hospital discharge.

**Objective:**

To evaluate the implementation of P5VS in public and private hospitals.

**Methods:**

Data analysis on validated questionnaires was sent to all 171 Portuguese hospitals via an official letter.

**Results:**

When compared to private hospitals, public hospitals presented a higher adherence to the process related to the P5VS. It has demonstrated superiority in the charts properly placed to record P5VS, in the number of emergency departments recording P5VS, in the regularity of audits, and in the existence of guidelines and staff training on pain assessment and management.

**Conclusion:**

The standardization of both evaluation and recording of pain intensity constitutes a measure of good clinical practice. Public hospitals demonstrated better commitment to these procedures that should be properly carried out in all health care institutions.

## Introduction

Pain assessment may be a challenging task due to its subjectivity since several factors can contribute to its underreporting. However, adequate pain management requires a well-documented assessment. Neglecting pain assessment and recording has been reported as the main cause of undertreated pain [1–4]. The assessment of pain as the fifth vital sign (P5VS) is of paramount importance since it leads to the management of undertreated pain, consequently reducing suffering, readmissions and emergency department visits after hospital discharge. It is important to be aware that P5VS should not be turned only into a bureaucratic procedure, and the process must include staff education and audits to ensure its accomplishment, avoiding both under and overtreatments [2, 5–10].

Historically, the former president of the American Pain Society, James Campbell, introduced the idea of “Pain as the 5^th^ Vital Sign” (P5VS) in 1996. According to this concept, pain intensity should be regularly assessed, together with the classic four vital signs. However, unlike the classic four vital signs, self-reporting of pain is a subjective measurement, and clinicians should accept patient reports and act upon them. Three years later, the P5VS campaign credibility increased substantially after being adopted by the Veteran’s Health Administration. In the same year of 1999, Assembly Bill 791 (Thomson) was signed in California to implement the assessment and prompt treatment of pain, it is recorded in the patient’s chart in a manner consistent with other vital signs [2, 11–13]. Likewise, the standards of pain assessment, follow-up, and uniform measurement were introduced as patients’ rights by the Joint Commission on Accreditation of Healthcare Organization (JCAHO) in 2000. This fact was followed by the North American pain management guidelines updates, including recommendations for pain assessment and documentation [3, 7, 14].

In 2003, the Portuguese Directorate-General for Health (DGH) published the directive number 09/DGCG, which established P5VS [4]. Within this framework, the regular evaluation and recording of P5VS were recognized as a good clinical practice, being mandatory in all health care services, together with the four classical vital signs, following the American Pain Society recommendations. With this regulation, which also includes guidelines to perform pain assessment, Portugal became the first country in the European Union to implement P5VS in the healthcare system.

Six years after the establishment of the National directive 09/DGCG, the present research was carried out by the National Observatory for Pain—NOPain to assess the implementation of P5VS all over the country, and to compare public and private healthcare systems.

## Methods

The present study followed the STROBE guidelines for cross‐sectional studies, being an analytical observational study. A questionnaire was developed by the NOPain team to appraise the compliance with the National directive 09/DGCG—Pain as the Fifth Vital Sign (Table 1). A pilot study was conducted by two nurses (department supervisors) and four physicians (one medical researcher and three anaesthetists) that suggested only minor reformulations in some statements for the final version. The gathering of data was divided into two phases. In the first phase, an official Portuguese State letter (sent and sealed by the Directorate-General for Health and Central Administration of the Health System) was sent to all Portuguese hospitals (public and private healthcare systems), addressed to the Hospital Administration and respective Ethics Committee. The letter included a detailed description of how to collect the data related to P5VS; the questionnaire; and a letter of acceptance to be filled by the Hospital Administration and the Ethics Committee of each participating hospital. The memorandum described that the study was approved and supported by the National Program against Pain of the Directorate-General for Health and Central Administration of the Health System. Since these documents were sent by an official governmental department for healthcare, and not by the researchers, acceptance was very high. Additionally, sample size calculation was not necessary because all Portuguese hospitals, with no exception, were invited to participate in this study.

**Table 1:** Questionnaire of P5VS implementation.

1—Is there a proper place in the hospital chart to record the patients’ pain intensity as the fifth vital sign? If yes, please list all the used pain scales.
2—In this hospital, what is the current percentage of departments that record pain as the fifth vital sign to all surgical patients?
3—Specifically in emergency care, is pain always recorded as the fifth vital sign?
4—In this hospital, is there a regular evaluation of the quality of the pain records in the hospital charts?
5—Is there written orientations distributed through the departments on how to proceed with the patients’ pain recording? If yes, could you please send us a copy?
6—In the last 3 years, did the hospital administration provide training on pain assessment to the nurse staff? If yes, which was the percentage of participation?
7—We kindly ask you to send us a copy of the hospital chart for surgical patients.
8—Identification: Name, Position, Hospital.

In the second phase, fifteen days after the letter was sent, a new personalized contact was made by the Central Administration of the Health System’s secretary to those hospitals that did not reply to the first request. The scope was to certify that the letter was received and examined. For those hospitals that did not receive it, a new letter was sent. Alternatively, for those hospitals that required it, all the information, including the questionnaire, was sent by electronic mail. To facilitate the answers, a fax number, an electronic mail address and a postal address were provided as well. Data from the received questionnaires were sent from the Central Administration of the Health System to the researchers of this study and inserted into a database (IBM SPSS Statistics 19.0) so that the statistical analysis could be performed.

Statistical analysis of P5VS implementation was carried out by estimating the proportion of hospitals that reported having a proper place to record patients’ pain intensity in hospital charts. Giving the hypothesis and aims previously stated, descriptive analysis of the frequencies and inferential statistics through Chi-square or Fisher exact tests were considered appropriate for the comparisons between public and private healthcare systems and a 95% confidence level was used.

The present study was approved by the Directorate-General for Health and Central Administration of the National Health System and followed the STROBE checklist for observational studies. Hospital Administrations and their respective Ethics Committees were individually addressed, approving this study when they agreed to complete and submit the questionnaires.

## Results

In the present study, all the 171 Portuguese hospitals were contacted, including 38 single hospitals and other 68 hospitals that were grouped in 25 National Health System (NHS) hospital centres. In addition, 65 private hospitals were also invited to participate. A total of 109 hospitals (S1 Table) completed the questionnaires, representing an overall response rate of 63.7%; 31 questionnaires were from private hospitals (47.7%) and 78 from the NHS (73.6%).

The questionnaires were filled by the head nurse (63.0%), by the nurse supervisor (13.0%), by the supervisor anaesthesiologist (8.3%), by the head nurse advisor or nurse coordinator (6.5%), by the clinical director (4.6%), by the general nurse (2.8%) and by the chairman of the hospital board (1.9%).

A comparative analysis between the NHS and private hospitals was conducted (Table 2). The results demonstrated an overall of 89.9% of the Portuguese hospitals having an adequate specifically devised place in the hospital charts to record P5VS. This was the case for 94.9% of the NHS hospitals, but only 77.4% of the private hospitals presented a proper place for pain registration (p = 0.012).

**Table 2:** Comparative analysis between public (NHS) and private hospitals (n = 109).

Question	NHS (n = 78)	Private (n = 31)	p* value
1—Is there a proper place in the hospital chart to record patients’ pain intensity as the fifth vital sign? n (%)			
No	4 (5.1)	7 (22.6)	**0**.**012**
Yes	74 (94.9)	24 (77.4)	
2—In your hospital, what is the current percentage of departments that record pain as the fifth vital sign in all patients? n (%)			
<25%	6 (7.9)	3 (11.5)	0.754
25 a 50%	9 (11.8)	4 (15.4)	
50 a 75%	18 (23.7)	4 (15.4)	
75 a 99%	28 (36.8)	8 (30.8)	
100%	15 (19.7)	7 (26.9)	
3—Specifically in the emergency department, is pain always recorded as the fifth vital sign? n (%)			
No	26 (38.3)	14 (82.4)	**0**.**001**
Yes	42 (61.8)	3 (17.6)	
4—In your hospital, is there a regular evaluation of the quality of the pain records in the hospital charts? n (%)			
No	50 (64.1)	24 (82.8)	**0**.**049**
Yes	28 (35.9)	5 (17.2)	
5—Are there written orientations distributed through the departments on how to proceed with patients’ pain recording? n (%)			
No	24 (31.2)	15 (50.0)	0.056
Yes	53 (68.8)	15 (50.0)	
5.1—If yes, could you please send us a copy? n (%)			
No	8 (15.1)	9 (60.0)	**0**.**001**
Yes	45 (84.9)	6 (40.0)	
6—In the last 3 years did your hospital provide training on pain assessment to the nurse staff? n (%)			
No	11 (14.1)	15 (50.0)	**0**.**001**
Yes	67 (85.9)	15 (50.0)	
6.1—If yes, which was the percentage of participation?			
<25%	6 (7.7)	2 (6.7)	**0**.**002**
25 a 50%	20 (25.9)	7 (23.3)	
50 a 75%	9 (11.5)	3 (10.0)	
75 a 99%	30 (38.5)	3 (10.0)	
100%	2 (2.6)	0 (0)	
Not applicable	11 (14.1)	15 (50.0)	
7—We kindly ask you to send us a copy of the hospital chart for surgical patients. n (%)			
No	44 (56.4)	18 (58.1)	0.482
Yes	34 (43.6)	13 (41.9)	

* Chi-square test or Fisher exact as appropriate.

The most used scale for evaluating pain intensity in the hospitals was a numeric rating scale (81.6%). Other scales reported to be used either alone or in combination with other scales for pain intensity assessment in the hospitals were the face scale (64.3%) and the visual analogue scale (43.9%). Additionally, most of the hospitals (73.5%) reported using combinations of scales, selecting the proper one according to the patient. In 78.4% of the cases, pain intensity was assessed and recorded in the majority of the hospital departments. However, only 21.6% of the hospitals applied it to all departments. The percentage of the hospitals with emergency departments recording P5VS was 52.9%. A remarkable difference between the NHS (61.8%) and private hospitals (17.6%, p = 0.001) was observed (Table 2).

The quality of the clinical charts was regularly audited in only 30.8% of the hospitals, being also higher in NHS (35.9%) than in private hospitals (17.2%, p = 0.049). Condensing the results of those audits, it was possible to observe differences ranging between 30 to 100% in the regular implementation of P5VS. Written guidelines for pain management were present in 63.6% of the Portuguese hospitals (68.8% of the NHS and only 50.0% of the private hospitals; p = 0.056). Staff training on pain assessment and management was promoted in 75.9% of the hospitals, being higher in NHS hospitals (Table 2).

## Discussion

The present study demonstrated that most of the Portuguese hospitals reported applying the DGH directive concerning P5VS [4]. This accomplishment followed North American pain management guidelines, making pain a priority regarding assessment and documentation [3, 7]. However, when compared to NHS hospitals, few private hospitals complied with this National directive.

The main goal for the implementation of P5VS is to assess and manage undertreated pain, consequently reducing the patient’s suffering and its associated healthcare costs. Adequate pain assessment and control may also help to reduce surgical readmissions and emergency department visits after hospital discharge [5, 8, 9, 15]. However, some of the results in the literature are contradictory. A study found that routinely measuring pain intensity may not increase the quality of pain management [16]. However, this study was performed in a single clinic, and the pain was managed based only on its intensity scores. On the contrary, several studies demonstrated that after the proper implementation of P5VS, the number of patients reporting moderate to severe pain significantly decreased [8, 17–19]. Nevertheless, introducing P5VS may not be enough if some principles of practice are not implemented, carried out and audited to effectively improve the patient’s health. A starting point would be to make pain assessment visible in hospital charts and to promote staff education on adequate pain assessment and prompt management, mainly during the postoperative period [2, 6–9, 20]. The present investigation showed that almost all NHS hospitals provided the staff with pain education and written information, hopefully contributing to pain management improvement, as reported in internal audits.

The use of pain scales intends to facilitate the communication between patients and staff, in an attempt to overcome this highly subjective and individual experience. The NRS, face and VAS scales, were the most used scales in Portuguese hospitals, being a reasonable tool for use in routine pain screening. However, pain scales should be carefully used because nurses may unintentionally fade into a bureaucratic checklist item filling, rather than enhancing communication [2, 10]. Special care should be taken to avoid inadequate pain assessment and management, or even lack of documentation on the subject [21–27], and the best concordance in pain reported by the patients and that registered by staff are related to the consistency of the standardized pain measures [20]. It was reported that some nurses may not be aware of the extension of pain recording, overestimating the quality of their documentation in the postoperative pain data [22, 24].

The use of reliable and validated scales can improve routine pain management. However, it is known that in the case of unmanaged postoperative pain, the main causes are insufficient multidimensional pain assessment, delays between assessment and analgesic administration, inadequate use of analgesics (under or overmedication) and poor communication between clinicians and patients [2, 7, 9, 22, 26, 28–30]. Thus, training, ongoing education programs based on the latest scientific evidence and feedback to nurses on their practice should also be considered when it comes to overcoming the described limitations such as lack of pain assessment and inadequate analgesia management [6, 7, 20–22, 27, 31]. In this context, hospital audits are necessary to evaluate and re-evaluate, if any of these limitations are jeopardizing pain assessment and treatment [8, 15]. In the present investigation, it was found that regular evaluation of the pain record quality was performed in only one-third of the hospitals.

Taking into account that pain is the most common reason for seeking medical attention and that acute non treated pain can lead to chronic pain, the concept of P5VS is paramount since it increases our understanding of pain, improving its assessment and relief [2, 7, 9, 13, 32]. However, pain assessment should be combined with the enforcement of existing guidelines for the management of acute pain to avoid overmedication, mainly by excessively using opioids [7, 13, 30]. It should be noted that opioid overuse has not been reported in Portugal [33, 34] and that an opioid epidemic is probably related to poor treatment choices and not to routine pain monitoring.

### Limitations

Despite the audits, one limitation of this study is the fact that what was written and reported by the hospitals may not be always applied in daily hospital practice. The fact that more NHS hospitals responded to the survey than private hospitals should also be considered a limitation in the comparisons made. Another potential source of bias is that those who answered the questionnaires were more likely to have implemented the P5VS in their hospitals, leading to an overestimated frequency of proper places in the hospital charts to record P5VS. Finally, the lack of more knowledge about local conditions in each hospital does not allow for a complete understanding of the whole process of P5VS implementation.

## Conclusion

To conclude, pain assessment as the 5^th^ Vital Sign, when properly used, allows monitoring the patient’s pain, contributing with proper strategies in pain management, fostering earlier mobilization, shortening hospital stay, increasing patient satisfaction and reducing costs [5–9, 17–19]. Evaluation and recording standardization of the patients’ pain intensity constitutes a measure of good clinical practice. Public hospitals demonstrated better commitment to these procedures that should be properly carried out in all health care institutions.

## Supporting information

S1 Table. Participating hospitals/institutions (DOCX)

S1 Data Data from this study. (XLSX)
