# Supplementary material for: Pain as the fifth vital sign—A comparison between public and private healthcare systems
Source: PLoS One. 2021 Nov 3;16(11):e0259535. doi: 10.1371/journal.pone.0259535 (PMC8565736; doi:10.1371/journal.pone.0259535)
Supplement: S1 Table. Participating hospitals/institutions — (DOCX) [file pone.0259535.s001.docx]

**S1 Word Table File. Participating hospitals/institutions.**

| **Participating Hospitals/Institutions** | **Type** |
| --- | --- |
| Hospital José Joaquim Fernandes | Public |
| Hospital de São Paulo | Public |
| Centro de Medicina e Reabilitação do Sul | Private |
| Centro de Medicina da Reabilitação da Região Centro | Private |
| Centro de Medicina de Reabilitação Alcoitão | Private |
| Centro Hospitalar Alto Minho - Hospital de Santa Luzia | Public |
| Centro Hospitalar Médio Tejo/Abrantes | Public |
| Centro Hospitalar Médio Tejo/Tomar | Public |
| Centro Hospitalar Médio Tejo/Torres Novas | Public |
| Centro Hospitalar Oeste Norte/Alcobaça | Public |
| Centro Hospitalar Oeste Norte/Caldas da Rainha | Public |
| Centro Hospitalar Oeste Norte/Hospital Termal Rainha Dona Leonor | Public |
| Centro Hospitalar Oeste Norte/PeniCentro Hospitalare | Public |
| Centro Hospitalar Psiquiátrico de Coimbra/Unidade de Arnes | Public |
| Centro Hospitalar Psiquiátrico de Coimbra/Unidade de Sobral Cid | Public |
| Centro Hospitalar Psiquiátrico de Coimbra/Unidade do Lorvão | Public |
| Clínica Central da Oiã | Private |
| Clínica da Associação | Private |
| Clínica de Montes Claros | Private |
| Clínica do Bom Jesus | Private |
| Clínica Europa | Private |
| Clínica Particular de Barcelos | Private |
| Clínica Todos-os-Santos | Private |
| Hospital Amato Lusitano | Public |
| Hospital Arcebispo João Crisóstomo | Public |
| Hospital Cândido de Figueiredo | Public |
| Hospital Comendador Manuel Moreira de Barros | Public |
| Hospital Conde de São Bento de Santo Tirso | Private |
| Hospital Curry Cabral | Public |
| Hospital da Horta | Public |
| Hospital da Lapa do Porto | Private |
| Hospital da Luz | Private |
| Hospital da Misericórdia de Valpaços | Public |
| Hospital da Misericórdia de Vila do Conde | Private |
| Hospital da Prelada | Private |
| Hospital da Universidade de Coimbra | Public |
| Hospital de Cascais | Private |
| Hospital de Centro Hospitalaraves | Public |
| Hospital de Faro | Public |
| Hospital de Joaquim Urbano | Public |
| Hospital de Júlio de Matos | Public |
| Hospital de Lamego | Public |
| Hospital de Magalhães Lemos | Public |
| Hospital de Nossa Senhora da Ajuda | Public |
| Hospital de Pulido Valente | Public |
| Hospital de Reynaldo dos Santos | Private |
| Hospital de Santa Maria | Public |
| Hospital de Santa Marta | Public |
| Hospital de Santarém | Public |
| Hospital de Santo André | Public |
| Hospital de São Bernardo | Public |
| Hospital de São João de Deus | Public |
| Hospital de São José | Public |
| Hospital de São José de Fafe | Public |
| Hospital de São Teotónio de Viseu | Public |
| Hospital de Torres Vedras | Public |
| Hospital Distrital da Covilhã | Public |
| Hospital Distrital da Figueira da Foz | Public |
| Hospital Distrital de Águeda | Public |
| Hospital Distrital de Lagos | Public |
| Hospital Distrital de Pombal | Public |
| Hospital Distrital do Fundão | Public |
| Hospital do Barlavento Algarvio | Public |
| Hospital do Desterro | Public |
| Hospital do Divino Espírito Santo de Ponta Delgada | Public |
| Hospital Dona Estefânia | Public |
| Hospital dos Lusíadas | Private |
| Hospital Dr. Francisco Zagalo | Public |
| Hospital Dr. José Maria Antunes Júnior | Public |
| Hospital Dr. José Maria Grande de Portalegre | Public |
| Hospital Eduardo Santos Silva | Public |
| Hospital Espirito Santo de Évora | Public |
| Hospital Fernando Fonseca | Public |
| Hospital Garcia de Horta | Public |
| Hospital Infante Don Pedro de Aveiro | Public |
| Hospital José Luciano de Castro de Anadia | Public |
| Hospital Miguel Bombarda | Public |
| Hospital Narciso Ferreira de Riba d'Ave | Private |
| Hospital Nossa Senhora do Rosário | Public |
| Hospital Ortopédico de Sant'Ana | Private |
| Hospital Ortopédico Santiago do Outão | Public |
| Hospital Particular de Lisboa | Private |
| Hospital Peso da Régua | Public |
| Hospital Prisional São João de Deus | Private |
| Hospital Privado da Trofa | Private |
| Hospital Privado São Gonçalo de Lagos | Private |
| Hospital Residencial do Mar | Private |
| Hospital Saint Louis | Private |
| Hospital SAMS | Private |
| Hospital Santa Luzia de Elvas | Public |
| Hospital Santa Maria Maior | Public |
| Hospital Santo António dos Capuchos | Public |
| Hospital Santo Espírito dos Açores | Public |
| Hospital São João | Public |
| Hospital São João da Madeira | Public |
| Hospital São João de Deus | Private |
| Hospital São Miguel | Public |
| Hospital São Pedro | Public |
| Hospital São Sebastião | Public |
| Hospital Senhora da Oliveira | Public |
| Hospital Visconde de Salreu de Estarreja | Public |
| HospitalParticular do Algarve | Private |
| Inst. Oftalmologia Dr. Gama Pinto | Public |
| Instituto Português de Oncologia Francisco Gentil | Public |
| Instituto Português Oncologia de Coimbra | Public |
| Instituto Português Oncologia do Porto | Public |
| INTERCIR – Centro Cirúrgico de Coimbra | Private |
| Santa Casa da Misericórdia do Entroncamento | Private |
| Unidade Local de Saúde de Matosinhos | Public |
